# Burst Firing in the Electrosensory System of Gymnotiform Weakly Electric Fish: Mechanisms and Functional Roles

**DOI:** 10.3389/fncom.2016.00081

**Published:** 2016-08-02

**Authors:** Michael G. Metzen, Rüdiger Krahe, Maurice J. Chacron

**Affiliations:** ^1^Department of Physiology, McGill UniversityMontreal, QC, Canada; ^2^Department of Biology, McGill UniversityMontreal, QC, Canada

**Keywords:** burst firing, weakly electric fish, feature detection, directional selectivity, neural coding, envelope

## Abstract

Neurons across sensory systems and organisms often display complex patterns of action potentials in response to sensory input. One example of such a pattern is the tendency of neurons to fire packets of action potentials (i.e., a burst) followed by quiescence. While it is well known that multiple mechanisms can generate bursts of action potentials at both the single-neuron and the network level, the functional role of burst firing in sensory processing is not so well understood to date. Here we provide a comprehensive review of the known mechanisms and functions of burst firing in processing of electrosensory stimuli in gymnotiform weakly electric fish. We also present new evidence from existing data showing that bursts and isolated spikes provide distinct information about stimulus variance. It is likely that these functional roles will be generally applicable to other systems and species.

## Introduction

Understanding how neurons process incoming sensory information thereby generating behavioral responses (aka the neural code) remains a central problem in neuroscience. While early neurophysiological studies assumed that neurons could only transmit information through changes in firing rate (i.e., a rate code; Adrian, 1941), more recent studies have shown that information can also be carried by precise spike timing using so-called temporal codes (Carr and Konishi, 1990; Bair, 1999; Panzeri et al., 2001; Johansson and Birznieks, 2004; Jones et al., 2004; Uzzell and Chichilnisky, 2004; Butts et al., 2007; Sadeghi et al., 2007; Mackevicius et al., 2012; Harvey et al., 2013; Saal et al., 2015; Zuo et al., 2015). Indeed, neurons often display complex intrinsic dynamics that influence their responses to sensory input. One example of such dynamics is the tendency of neurons to fire packets of action potentials (i.e., bursts) followed by quiescence, which is seen ubiquitously in the central nervous system (for review see Krahe and Gabbiani, 2004). While the mechanisms that lead to burst firing are generally well understood (Llinas and Jahnsen, 1982; Huguenard and Prince, 1992; Wang and Rinzel, 1995; Azouz et al., 1996; Magee and Carruth, 1999; Schwindt and Crill, 1999; Izhikevich, 2000; Lemon and Turner, 2000; Su et al., 2001; Doiron et al., 2003b; Noonan et al., 2003), their role in information processing, despite decades of research on the subject, is still a matter of debate (Crick, 1984; Lisman, 1997; Krahe and Gabbiani, 2004; Gollisch and Meister, 2008; Marsat and Pollack, 2012).

Here we review recent advances towards understanding the functional role of burst firing in a model system benefiting from well-characterized neural circuits as well as the use of naturalistic stimuli, the electrosensory system of the gymnotiform wave-type weakly electric fish *Apteronotus leptorhynchus* (Chacron et al., 2011; Marsat et al., 2012; Krahe and Maler, 2014; Clarke et al., 2015). These fish sense perturbations of their self-generated electric organ discharge (EOD) through an array of peripheral electroreceptor afferents (EAs) that synapse onto pyramidal cells within the electrosensory lateral line lobe (ELL). These pyramidal cells in turn synapse onto neurons within the midbrain torus semicircularis (TS). Natural stimuli for weakly electric fish are well characterized and consist of amplitude modulations (AMs) of the EOD and are discussed in detail below.

The article is organized as follows. After a brief presentation of the relevant circuitry and natural stimuli, we review potential mechanisms that give rise to burst firing in EAs. In particular, EAs can be segregated into two subpopulations: bursting and tonic. We review proposed functional roles for each subpopulation. We then review how interactions between the soma and dendrites mediate burst firing in ELL pyramidal cells. Accumulating evidence suggest that such burst firing signals the presence of specific features of natural stimuli. We next focus on TS neurons, for which burst firing mediated by calcium channels can more reliably signal the direction of a moving object than the full spike train. We then present the results of new analyses of previously published data showing that burst firing in peripheral EAs can enhance the neuronal gain to stimulus contrast, which is similar in concept to the function described in TS. We finish by drawing some general conclusions about the functional roles of burst firing in the electrosensory system.

## Background

### The Electrosensory System of *Apteronotus leptorhynchus*

**Figure 1:**
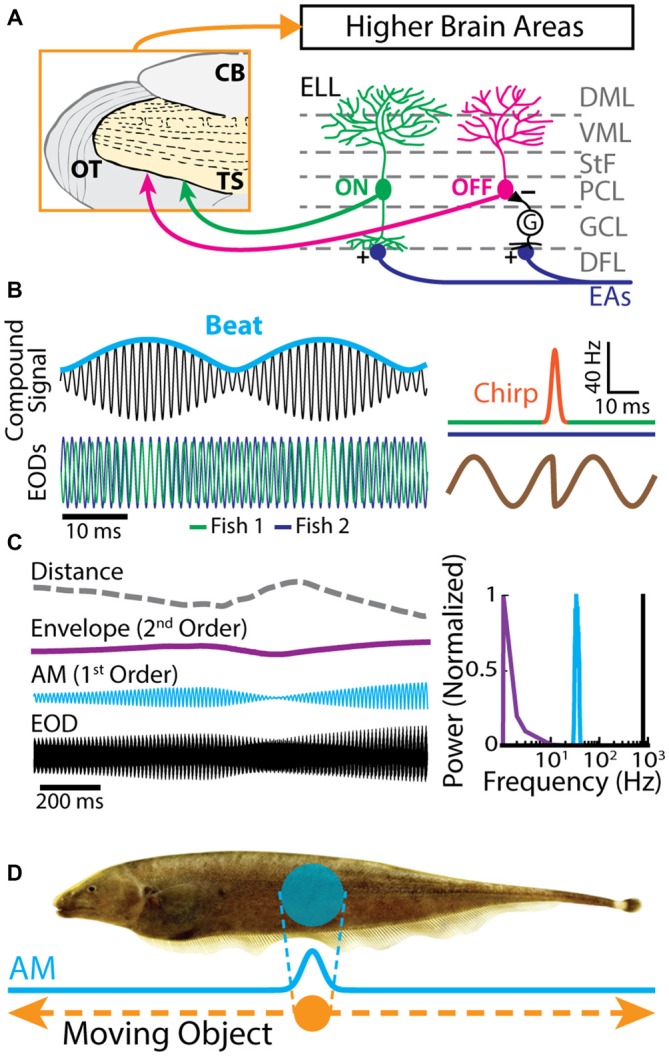
**Electrosensory circuitry and natural stimuli. (A)** Peripheral electrosensory afferents (EAs) enter the hindbrain at the deep fiber layer (DFL) of the electrosensory lateral line lobe (ELL) and project onto two types of pyramidal neurons (ON: green; OFF: magenta) within the pyramidal cell layer (PCL). ON type cells have a basilar dendrite that connects directly to the EAs, while OFF type cells lack such a basilar dendrite and instead receive disynaptic input via local interneurons (*G*) within the granule cell layer (GCL). The apical dendrites of both types extend through the stratum tractus fibrosum (StF) to the molecular layers of the ELL (VML, ventral molecular layer; DML, dorsal molecular layer). Both types of neurons send projections to higher brain areas, such as the midbrain torus semicircularis (TS). **(B)** *Left*: the electric organ discharges (EODs) of two fish (green and blue) interfere and thus create a sinusoidal beat (cyan) whose frequency is equal to the EOD frequency difference between the two fish. *Right*: during an electro-communication call (i.e., a chirp), the emitter fish’s EOD frequency (top green trace) transiently increases for a brief period of time (top orange trace), while the receiver fish’s EOD frequency (top blue trace) remains constant. The chirp results in a phase reset of the beat (bottom brown trace). **(C)** *Left*: EOD waveform from *Apteronotus leptorhynchus* (black) with amplitude modulation (AM, cyan) and envelope (purple) waveforms. We note that the envelope corresponds to the depth of modulation of the EOD AM that is due to relative movement (dashed gray line) between individuals. *Right*: shown are the frequency contents of the full signal (black), the AM (cyan), and the envelope (purple). **(D)** EOD AM (cyan) originating from an object (orange) that is moving along the fish’s body (dashed orange arrows) and the corresponding electric image projected onto the skin (cyan).

The gymnotiform weakly electric fish *Apteronotus leptorhynchus* produces an electric field surrounding its body by generating the EOD. This electric field is used for electrolocation purposes as well as during electro-communication with conspecifics. Objects with conductivity other than the surrounding water or interference with the EODs of conspecifics perturb the transdermal potential established by the fish’s EOD. EAs in the skin sense AMs of the EOD (Chacron et al., 2011; Marsat et al., 2012; Krahe and Maler, 2014). In general, EAs increase their firing rates with increasing EOD amplitude (Scheich et al., 1973). Each EA furthermore projects topographically onto pyramidal neurons located within the hindbrain ELL (Heiligenberg and Dye, 1982; Krahe and Maler, 2014). The ELL is organized in layers: a deep fiber layer, DFL, where EAs terminate, layers of GABAergic interneurons and pyramidal cell somata (granule cell layer, GCL; pyramidal cell layer, PCL), and molecular layers (ventral molecular layer, VML; dorsal molecular layer, DML) containing the apical dendrites of pyramidal cells (Figure 1A). There are two main classes of pyramidal neurons: ON- and OFF-cells (Figure 1A, right). ON-cells have basal dendrites that receive direct excitatory synaptic input from EAs and thus respond with increases in firing rate to increases in EOD amplitude. OFF-cells on the other hand receive di-synaptic inhibitory input from EAs via local interneurons and will thus respond with increased firing rate to decreases in EOD amplitude (Maler, 1979; Maler et al., 1981). ON and OFF type pyramidal cells are the sole output of the ELL and project to TS, a layered structure within the midbrain of these fish (Figure 1A, left; Maler, 1979; Bastian et al., 2004) that is the equivalent of the mammalian inferior colliculus.

### Behaviorally Relevant Electrosensory Stimuli

Natural electrosensory stimuli consist of sinusoidal variations in the amplitude of each fish’s own EOD that arise in multiple behaviorally relevant contexts. For example, when two fish are located close to one another (i.e., <1 m), interference between their EODs will cause a sinusoidal AM (i.e., a beat, whose waveform is considered a first-order stimulus attribute, Figure 1B) at temporal frequencies of 0–400 Hz depending on the EOD frequency difference between the two fish (Hupé and Lewis, 2008). Moreover, brief increases in EOD frequency known as “chirps” are used as electro-communication signals and transiently perturb the beat pattern (Figure 1B, right; Benda et al., 2005; Hupé and Lewis, 2008; Marsat and Maler, 2010; Aumentado-Armstrong et al., 2015; Metzen et al., 2016). On the other hand, the beat amplitude (i.e., the envelope, a second-order stimulus attribute) is modulated when fish move relative to one another (Figure 1C, purple line; Stamper et al., 2012; Yu et al., 2012; Huang et al., 2016; Zhang and Chacron, 2016). As such, the envelope depends on the relative distance and orientation between animals (Figure 1C, gray dashed line; Yu et al., 2012). Indeed, if two fish are in close proximity to one another, the envelope is high. In contrast, the envelope is low when the fish are located farther apart from one another (Figure 1C, left). The envelope typically varies slowly in time and thus contains temporal frequencies of less than 1 Hz (Figure 1C, right; Yu et al., 2012; Fotowat et al., 2013; Stamper et al., 2013; Metzen and Chacron, 2014).

The previously described stimuli are spatially diffuse as they impinge on most if not all of the animal’s skin surface. In contrast, spatially localized EOD AMs can occur if objects such as prey move along the fish’s body (Figure 1D; Bastian, 1981b; Chacron et al., 2003a, 2011; Chacron and Fortune, 2010). While EAs are not sensitive to the stimulus’ spatial extent as long as it impinges upon their receptive fields (Chacron et al., 2005c), ELL pyramidal cells can show a strong dependence on the stimulus’ spatial structure due to interactions between the center and surround portions of their receptive fields (Chacron et al., 2003a). Further, neither EAs nor ELL pyramidal cells show sensitivity to the direction of movement (i.e., they are not directionally selective; Bastian, 1981b; Chacron et al., 2009, 2011), which is not the case for TS neurons (Chacron et al., 2009; Chacron and Fortune, 2010; Khosravi-Hashemi et al., 2011; Khosravi-Hashemi and Chacron, 2012) as discussed below.

## Burst Firing in the Electrosensory System

### Burst Firing in Peripheral Afferents

**Figure 2:**
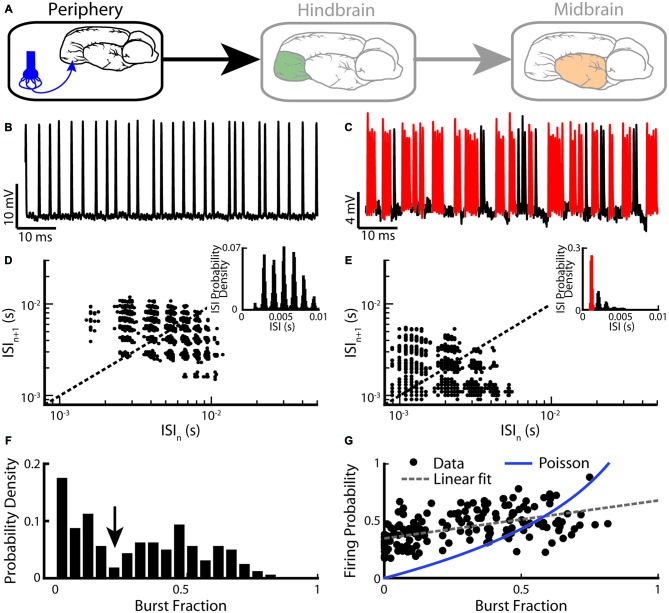
**Electrosensory afferents (EAs) are composed of two sub-populations: bursting and non-bursting. (A)** Primary afferents from peripheral electroreceptors project onto pyramidal neurons within the hindbrain. **(B)** Example recording of a non-bursting EA. **(C)** Example recording from an EA that displays burst firing (red). **(D)** Return map of the same neuron shown in **(B)**. *Inset*: interspike interval (ISI) distribution. **(E)** Return map of the same neuron shown in **(C)**. *Inset*: ISI distribution. **(F)** Segregating a population (*n* = 94) based on burst fraction (i.e., fraction of ISIs below a threshold corresponding to the inverse of the EOD frequency indicated by the arrow ~2 ms) reveals two subpopulations of EAs (Two-sample Kolmogorov-Smirnov test, *p* ≪ 10^–3^). **(G)** Plotting firing probability as a function of burst fraction yields a positive correlation (*r* = 0.74). Also shown is the firing probability as a function of burst fraction for an equivalent Poisson process (blue curve). The data plotted in **(B–G)** are from Metzen and Chacron (2015).

We first describe burst firing at the sensory periphery (Figure 2A). EAs display strong heterogeneities in their baseline activity (i.e., in the absence of stimulation but in the presence of the animal’s unmodulated EOD). Indeed, their baseline firing rates range between 150 Hz and 600 Hz (Nelson et al., 1997; Gussin et al., 2007; Metzen and Chacron, 2015; Metzen et al., 2015b). It is well known that the firing rate of an EA increases as a function of EOD amplitude and is limited by the EOD frequency as EAs can fire at most one action potential during each EOD cycle (Scheich et al., 1973; Bastian, 1981a; Xu et al., 1996). More recent studies have focused on action potential patterning: while some EAs fire in a tonic manner (Figure 2B), others instead fire clusters of action potentials (i.e., bursts) followed by quiescence (Figure 2C; Bastian, 1981a; Xu et al., 1996). All EAs display phase locking to the animal’s quasi-sinusoidal EOD as action potentials preferentially occur near a local EOD maximum. Upon closer inspection, it is seen that a random number of EOD cycles occurs between two consecutive action potentials for the tonically firing EA (Figure 2B). In contrast, action potentials within a burst tend to occur on consecutive EOD cycles for the bursting EA (Figure 2C). The fact that EA spike trains are phase-locked to the EOD implies that interspike intervals (ISIs) tend to cluster near integer multiples of the EOD period. Thus, ISI histograms (ISIHs) from EAs are multimodal with each mode centered on an integer multiple of the EOD period (Figures 2D,E, insets). However, only the ISIH obtained from the bursting EA shows a prominent peak near the EOD period (compare insets of Figures 2D,E). As such, bursts of action potentials can be identified using an ISI threshold that is set to 1.5 EOD cycles. Only action potentials separated by an interval less than the burst threshold are deemed to be part of a burst.

Burst firing in EAs can be also investigated by plotting the return map (i.e., the current ISI as a function of the preceding ISI). While the return map obtained from the tonic EA showed clusters around the identity line (Figure 2D), that obtained from the bursting EA showed clusters indicating that short ISIs are followed by long ISIs and vice versa (Figure 2E), which is required for burst firing (Xu et al., 1996; Chacron et al., 2000, 2001b). Burst firing in EAs has been quantified by computing the burst fraction (i.e., the fraction of ISIs whose value is less than the burst threshold). Interestingly, the distribution of burst fractions within the EA population is bimodal (Kolmogorov-Smirnov test, *p* ≪ 10^−3^; Figure 2F). This implies that there are two distinct EA sub-populations: one with low burst fraction (i.e., “tonic”) and the other with high burst fraction (i.e., “bursting”). Moreover, the firing probability across the EA population is positively correlated with the burst fraction (Figure 2G, *r* = 0.74), indicating that bursting electroreceptors tend to display higher baseline firing rates than their tonic counterparts. Burst fractions for EAs were less than that of a Poisson process with the same firing rate when low (<0.4) values were considered (Figure 2G). This is not unexpected as EAs display strong refractoriness that limits the fraction of ISIs below the burst threshold. In contrast, for higher burst fraction values (>0.4), burst fractions of EAs were more or less equal to that of an equivalent Poisson process, implying that the burst mechanism must facilitate action potential firing in order to compensate for refractoriness.

The mechanisms underlying burst firing in EAs have been investigated using mathematical models (Chacron et al., 2001a,b, 2004c; Benda et al., 2005, 2010; Savard et al., 2011). Specifically, a generic model based on the leaky integrate-and-fire formalism (Lapicque, 1907) has been proposed and described in detail elsewhere (Chacron et al., 2000, 2001a). Briefly, the membrane potential is integrated until it reaches the action potential threshold and a spike is said to have occurred. The membrane potential is then reset and the threshold incremented by a fixed amount. The threshold then decays between action potentials. This simple model showed surprising accuracy at reproducing the baseline spiking activities of tonic EAs (Chacron et al., 2000, 2001a,b). This model was furthermore accurate at reproducing the baseline spiking activities of bursting EAs with a simple modification that involved the addition of a depolarizing current after each action potential (Chacron et al., 2000, 2001b, 2004c). Thus, mathematical models predict that burst firing in EAs is due to excitatory currents occurring after each action potential, thereby promoting further action potential firing. Burst firing is eventually terminated because of accumulation of refractoriness during a burst (modeled by cumulative increases in the action potential threshold under repetitive firing). These predictions remain untested to this day.

The functional role of spike patterns in EAs has been investigated in previous studies. The strong patterns found in their baseline activities as reflected by negative correlations between successive ISIs have been shown to improve their ability to detect weak signals such as those caused by prey objects (Chacron et al., 2001a, 2003b, 2005b) through noise reduction (Chacron et al., 2004b, 2005a; for review, see Chacron et al., 2004a; Ávila-Akerberg and Chacron, 2011b). However, much less is known about the functional role of burst firing. A modeling study has compared the performances of tonic and bursting EAs at estimating the time course of the stimulus (i.e., stimulus estimation) vs. detecting specific stimulus features (i.e., feature detection). Confirming results from other systems (Sherman, 2001), it was found that the tonic model EA was best at stimulus estimation while the bursting model EA was best at feature detection (Chacron et al., 2004c). Thus, this modeling study predicts separate functional roles for the tonic and bursting EA subpopulations. This prediction has, however, not yet been tested experimentally. Another important functional role for burst firing in electroreceptors concerns the coding of natural communication stimuli. Indeed, natural electro-communication stimuli such as chirps can elicit synchronous burst firing from EAs (Benda et al., 2005) and a recent study has shown that such synchronous bursts are necessary in order for the animal to correctly perceive the same electro-communication chirp stimulus occurring under different contexts (Metzen et al., 2016).

Any information transmitted must of course be decoded by higher order neurons in order to be useful to the organism. Thus, we now briefly review some evidence showing that bursts of action potentials in EAs are actually decoded by their downstream targets: ELL pyramidal cells. Evidence that the time in between consecutive action potentials in EAs is important first comes from studies of synaptic plasticity at EA-ELL pyramidal cell synapses. Experiments conducted *in vitro* have found that these synapses display short-term depression that is matched to the statistics of EA spike trains in order to further reduce noise (Khanbabaie et al., 2010). ELL pyramidal cells also display subthreshold inward currents that facilitate action potential firing in response to synchronous EA bursts (Berman and Maler, 1999; Middleton et al., 2009).

To conclude this section, while it is well known that EAs can fire bursts of action potentials, the mechanisms underlying, and the functional role of burst firing are just beginning to be understood. There is, however, evidence that burst firing in EAs is decoded downstream and is thus of importance to the organism. We now review burst firing in ELL pyramidal cells.

### Burst Firing in the Hindbrain ELL

**Figure 3:**
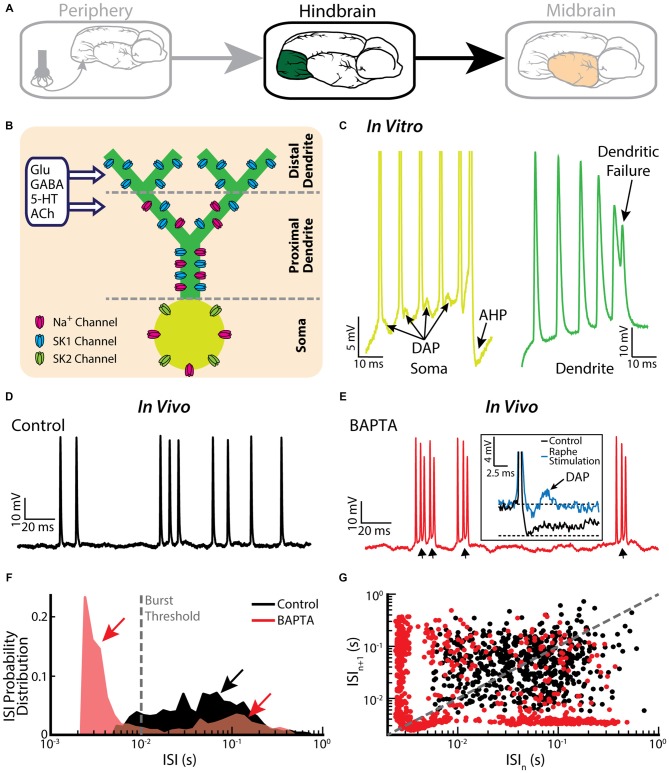
**Bursting in neurons in the hindbrain ELL. (A)** ELL pyramidal cells receive input from the electrosensory primary afferents and project to the midbrain.** (B)** Schematic showing the distribution of sodium (Na^+^, magenta), and two subtypes of small-conductance potassium (blue: SK1; green: SK2) channels. Na^+^ channels are located in the soma as well as the proximal dendrite, SK1 channels are located in the proximal and distal dendrite, whereas SK2 channels are only expressed in the soma. Neuromodulators, such as serotonin (5-HT) and acetylcholine (ACh), influence spiking. **(C)** A somatic and dendritic burst of spikes recorded separately in two cells (somatic spikes are truncated). The slowdown in dendritic spike repolarization is due to inactivation of a dendritic K^+^ conductance and results in a potentiation of the somatic depolarizing afterpotential (DAP; arrows). When the DAP reaches threshold for a high-frequency spike doublet, the second spike fails to backpropagate. This allows the afterhyperpolarization (AHP) to terminate the burst. **(D)** Example *in vivo* recording of an ELL pyramidal cell under control conditions. **(E)** The same neuron as in **(E)** displays bursting after treatment with the Ca^2+^ chelator BAPTA. The arrows indicate the ramp depolarizations. *Inset*: a DAP is seen after electrically stimulating serotonergic pathways (blue line). **(F)** ISI distribution under control condition (black) and after BAPTA treatment (red) showing a decrease in the cell’s absolute refractory period and the emergence of a second peak (red arrows). The burst threshold used to segregate bursts and isolated spikes for ELL pyramidal cells was 10 ms. **(G)** ISI return map under control condition (black) and after BAPTA treatment (red) showing a transition to a bursting regime. The data plotted in **(D–G)** are from Toporikova and Chacron (2009).

Pyramidal cells within the hindbrain ELL also display burst firing (Figure 3A). The underlying burst mechanism is well understood and has been extensively studied (Turner et al., 1994; Lemon and Turner, 2000; Doiron et al., 2001, 2002; Laing and Longtin, 2003; Laing et al., 2003; Fernandez et al., 2005). The mechanism is intrinsic in nature and involves interaction between sodium channels located on the soma and on the proximal dendritic tree (Figure 3B). Figure 3C illustrates the mechanism: an action potential backpropagates to the dendrite, in turn causing a dendritic action potential that propagates back to the soma and causes a depolarizing afterpotential (DAP). The DAP depolarizes the membrane potential at the soma, thereby eliciting another somatic action potential. This “ping-pong” interaction between soma and dendrite continues throughout the burst. In particular, the DAP increases in amplitude during a burst, thereby leading to shorter ISIs. The burst is terminated when the ISI becomes shorter than the dendritic refractory period. Thus, the somatic action potential does not elicit another dendritic action potential (i.e., there is a “dendritic failure”) and no DAP is then seen at the soma. Instead, a large afterhyperpolarization (AHP) can be measured (Figure 3C). The tendency to fire a burst of action potentials increases with the state of depolarization of the soma. Interestingly, pyramidal cells also display a “burst threshold” below which they fire tonically and above which burst firing occurs (Doiron et al., 2003b). This mechanism is reminiscent of the dual modes of firing seen in thalamic relay neurons (Sherman, 2001).

It is important to note that the burst firing mechanism described above has been primarily investigated *in vitro*. While the baseline activities of pyramidal cells recorded *in vivo* also contain bursts, their structure is quite different than that observed *in vitro* (Bastian and Nguyenkim, 2001; Figure 3D), which has important consequences for understanding their functional role as described below. Burst firing after application of the calcium chelator BAPTA, however, resembles more closely burst firing seen under *in vitro* conditions (Toporikova and Chacron, 2009; compare Figure 3E with Figure 3C). While this result supports the hypothesis that the burst firing seen* in vivo* is also intrinsic in nature, the potential contribution of network mechanisms cannot be ruled out. Comparison of ISIHs obtained before and after BAPTA application reveals a bimodal distribution only in the latter case (Figure 3F), further confirming the different nature of the burst mechanisms (Turner et al., 1996; Mehaffey et al., 2008b). Further characterization by computing ISI return maps reveals an L-shaped cluster along the abscissa and the ordinate after BAPTA application (Figure 3G, red dots), which is similar to that seen *in vitro* (Ellis et al., 2007b). This L-shaped cluster is not present under control conditions (Figure 3G, black dots). Further modeling studies have explained how BAPTA can give rise to increased burst firing. Under control conditions, calcium-activated potassium (SK) channels (Figure 3B) give rise to an AHP after the spike, thereby opposing further action potential firing and bursting. SK1 channels located on the apical dendrites of both ON- and OFF-type pyramidal cells as well as SK2 channels located on the soma of ON-type pyramidal cells contribute to the AHP. Throughout the burst, both the DAP and the AHP increase but the AHP increases at a faster rate, thereby leading to an “early termination” of the burst (Toporikova and Chacron, 2009). BAPTA decreases the AHP amplitude, thereby “unmasking” burst firing that is similar to that seen *in vitro*.

Burst firing in ELL pyramidal cells is heavily regulated by feedback (Doiron et al., 2003a; Mehaffey et al., 2005) as well as by neuromodulators such as serotonin (Deemyad et al., 2011) and acetylcholine (ACh; Ellis et al., 2007a; Mehaffey et al., 2008a; for review see Marquez et al., 2013). In particular, application of serotonin enhances burst firing in part through inhibition of SK channels (Ellis et al., 2007b; Deemyad et al., 2011, 2012). While studies performed *in vitro* have shown that SK2 channels are inhibited by serotonin application (Deemyad et al., 2011), similar effects of serotonin application on both ON- and OFF-type pyramidal cells *in vivo* suggest that SK1 channels are also being inhibited. In both cases, serotonin increases pyramidal neuron excitability through a reduction of the spike AHP, thereby unmasking the DAP (Figure 3E, inset; Deemyad et al., 2013) in a similar fashion as shown for the BAPTA application. Furthermore, multi-unit recordings from ELL pyramidal cells have revealed that the spike trains of neighboring cells are not independent but are instead correlated with one another (Chacron and Bastian, 2008; Litwin-Kumar et al., 2012; Simmonds and Chacron, 2015). Such correlations are primarily caused by synchronous burst firing (Chacron and Bastian, 2008). Thus, much more is known about the mechanisms leading to burst firing in ELL pyramidal cells than those leading to burst firing in EAs.

What is the functional role of burst firing in the ELL? It has been suggested that burst firing in general can improve the signal-to-noise ratio (Sherman, 2001), thereby transmitting specific or additional information about a sensory stimulus (Reinagel et al., 1999; Keat et al., 2001; Kepecs et al., 2002; Martinez-Conde et al., 2002). When investigating the functional role of burst firing, it is critical to consider whether information is contained in its actual structure. This is important since burst length (i.e., the number of spikes within a burst) is predicted to code for stimulus slope by mathematical models (Kepecs et al., 2002). While studies have reported that the burst ISI could code for stimulus intensity in ELL pyramidal cells (Doiron et al., 2007; Oswald et al., 2007), these were conducted *in vitro*. However, *in vivo* studies of ELL pyramidal cells have found that bursts contained no significant information about the stimulus either in their length or in the timing of action potentials within the burst (Ávila-Akerberg et al., 2010; Ávila-Akerberg and Chacron, 2011a). For this reason, bursts in ELL pyramidal cells are for the most part assumed to represent events.

One of the first functional roles identified for burst firing in ELL pyramidal cells was to signal the presence of specific stimulus features. Indeed, the performance of ELL pyramidal cells at reconstructing the detailed time course of the stimulus is much lower than that of EAs (Gabbiani et al., 1996; Metzner et al., 1998; Bastian et al., 2002). Bursts of action potentials of pyramidal cells are, however, more reliable detectors of stimulus features (Gabbiani et al., 1996; Metzner et al., 1998). Further studies have shown that the features in question correspond to the low-frequency components of a stimulus (Oswald et al., 2004; Ávila-Akerberg et al., 2010; Middleton et al., 2011). Interestingly, those spikes that are not part of a burst (i.e., “isolated” spikes) instead code for the high-frequency components of a stimulus. A similar functional separation was found in thalamic relay neurons (Lesica and Stanley, 2004), suggesting that parallel processing of different stimulus attributes by bursts and isolated spikes is a general feature of sensory processing.

Another important functional role of burst firing is to regulate the plasticity of feedback inputs received by ELL pyramidal cells. Indeed, one important function for feedback is to regulate the gain of pyramidal cells to sensory input (i.e., gain control; Bastian, 1986a,b). This is important, as the animal must distinguish between sensory input that is caused by its own movements (i.e., re-afference) and sensory input caused by external sources (i.e., ex-afference), a very general problem that must be solved by every organism (Cullen, 2011). In gymnotiform wave-type weakly electric fish, the feedback consists of a negative image of the re-afferent stimulus (Bastian, 1999). When the re-afferent stimulus and the negative image are matched in amplitude, the ELL pyramidal cell will not respond to the re-afferent sensory input and will be able to respond selectively to ex-afferent input. Changes in the strength of the re-afferent input (e.g., those experienced during development) must be compensated by changes in the feedback input. Such changes in feedback are achieved through anti-Hebbian synaptic plasticity that is triggered by burst firing in ELL pyramidal cells (Harvey-Girard et al., 2010). Indeed, a response to re-afferent input will initially trigger burst firing, which will reduce the strength of excitatory feedback input, thereby reducing the cell’s response to re-afferent input (Bol et al., 2011, 2013). Burst firing in ELL pyramidal cells thus plays a similar functional role to that of complex spikes in mormyrid weakly electric fish (Roberts, 1999; Roberts and Bell, 2000).

Yet another functional role for burst firing is the coding of natural electro-communication stimuli such as chirps. In fact, the combined excitation resulting from chirps as well as that caused by the underlying beat can sometimes trigger bursts that are terminated by dendritic failure despite the presence of the AHP (Marsat et al., 2009; Marsat and Maler, 2012; for review see Marsat et al., 2012). At the population level, chirp stimuli are predicted to trigger synchronized burst firing in ELL pyramidal cells. This result is at first glance paradoxical since chirps can consist of high-frequency transients, and the studies cited above have shown that bursts instead code for the low-frequency components of the stimulus. It is, however, important to consider that burst firing in ELL pyramidal cells can have different structures as mentioned above. Under some conditions, the AHP leads to an early burst termination; under other conditions, burst firing terminates by a dendritic failure. It is burst firing of the former type that has been primarily considered in studies showing coding of the low-frequency components of the stimulus. In contrast, coding of chirp stimuli is achieved by burst firing of the latter type.

**Figure 4:**
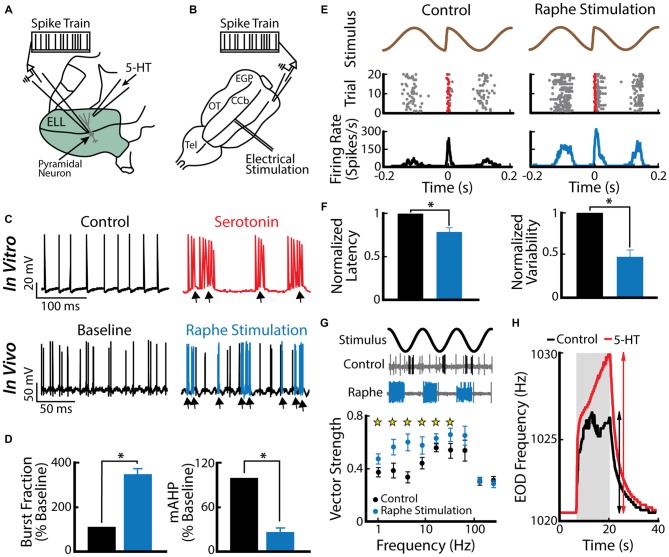
**Serotonin increases electrosensory pyramidal neuron excitability. (A)** Schematic showing the setup used to apply serotonin focally. Shown are the recording electrode that is positioned near a pyramidal neuron and the pipette containing serotonin that is positioned close to this neuron’s dendritic tree. **(B)** Schematic showing how stimulation of the raphe nuclei was achieved. Shown is a dorsal view of the animal’s brain with the recording pipette and the stimulation electrode. CCb, corpus cerebelli; EGP, eminentia granularis posterior; Tel, telencephalon; OT, optic tectum. **(C)** *Top*: spiking activity from an example ELL pyramidal neuron recorded *in vitro* under control conditions (left) and after serotonin application (right). Note that the application of serotonin induces burst firing (arrows). *Bottom*: spiking activity from an example ELL pyramidal neuron recorded *in vivo* under baseline (left) and raphe nuclei stimulation (right). Note the increased burst firing (blue) after raphe stimulation. **(D)** Left: population-averaged burst fraction (i.e., the fraction of ISIs < 10 ms) before stimulation (black), and after raphe stimulation (blue, *n* = 13). *Right*: release of serotonin through raphe stimulation led to a significant reduction in the medium component of the AHP (mAHP) in pyramidal neurons. Asterisks indicate statistical significance at the *p* = 0.05 level using a paired *t*-test. **(E)** Stimulus waveform showing a chirp at the center (top), raster plot (middle) showing spike times (gray), and the first spike occurring immediately after the small chirp (red) as well as the corresponding peristimulus time histogram (PSTH; bottom) before (left) and after (right) raphe stimulation. **(F)** Bar graphs showing the population-averaged normalized first spike latency (left, *n* = 13) and the normalized SD of the first spike latency (right, *n* = 13) before (black) and after raphe stimulation (blue). **(G)** Top: stimulus waveform, an example control recording of a pyramidal cell (burst spikes in black) and a recording from the same cell after raphe stimulation (burst spikes in blue). Bottom: Population-averaged vector strength values as a function of stimulus frequency before (black, *n* = 6) and after (blue, *n* = 6) raphe stimulation. **(H)** EOD frequency in response to a jamming stimulus as a function of time before (control, black) and after serotonin (serotonin, red) injection. Note the higher increase in EOD frequency after serotonin injection compared to the control condition. Data plotted are from Deemyad et al. (2013).

A recent study has shown that an important function for burst firing in ELL pyramidal cells is to signal stimulus features associated with a same-sex conspecific (Deemyad et al., 2013). Indeed, serotonin application within the ELL increased the detectability of electrosensory signals associated with a same sex conspecific (low frequency AMs as well as electro-communication signals such as chirps) by enhancing burst firing in ELL pyramidal cells (Deemyad et al., 2013). In this study, the authors applied serotonin focally on pyramidal neurons (Figure 4A) in addition to activating the serotonergic pathways by electrical stimulation of the raphe nucleus *in vivo* (Figure 4B). Both methods led to similar increased excitability and burst firing in pyramidal cells (Figure 4C, compare top and bottom). The authors further reported that the burst fraction of ELL pyramidal neurons was significantly increased after serotonin treatment (Figure 4D, left). Furthermore, the same study showed that serotonin significantly reduces the AHP (Figure 4D, right) and thus promotes burst firing through increased pyramidal neuron excitability. The authors suggested that serotonergic input selectively improves pyramidal cell responses to stimuli that occur during interactions between conspecifics, i.e., beats and chirps (Figures 4E,G). Serotonin release through raphe stimulation decreased spiking latency and increased spiking reliability to chirp electro-communication signals (Figure 4F). When using only AMs, but with different frequencies to mimic the beats that occur when two conspecific individuals come into close proximity to one another, raphe-triggered serotonin release significantly increased phase locking to low (<32 Hz), but not high-frequency beats (Figure 4G; Deemyad et al., 2013), which increases their perception by the animal (Figure 4H). Together, these findings suggest that the function of serotonin in the ELL is to selectively enhance the response of pyramidal neurons to stimuli that are generated during interactions between same-sex conspecifics that have similar EOD frequencies.

Burst firing in ELL pyramidal cells is likely to be functionally relevant as there is evidence that downstream neurons within the midbrain TS can respond to burst firing. In fact, some TS neurons respond selectively to chirp stimuli (Vonderschen and Chacron, 2011) and intracellular recordings suggest that this is because such stimuli give rise to a large depolarization that is not caused by the beat (Vonderschen and Chacron, 2009). The most parsimonious explanation is that these TS neurons respond to synchronous bursts from ELL pyramidal cells caused by the chirp stimulus. TS neurons also respond more selectively to stimuli than ELL pyramidal cells. In particular, some are tuned primarily to low temporal frequencies while others are instead tuned primarily to high frequencies (for review see Chacron et al., 2011). It is possible that such tuning arises in part because the former respond primarily to bursts of action potentials while the latter respond primarily to isolated spikes. Realistic neural circuits that will extract the bursts or isolated spikes have been proposed (Khosravi-Hashemi et al., 2011; Khosravi-Hashemi and Chacron, 2012) and could be implemented within TS. However, the most convincing evidence supporting the hypothesis that bursts in ELL pyramidal cells are functionally relevant is the fact that serotonin application within the ELL will give rise to increased perception of stimuli as measured from the animal’s behavioral responses (Deemyad et al., 2013).

Thus, burst firing within the ELL is likely to have multiple functional roles that involve feature detection. However, bursts can be elicited by multiple stimulus features, which could potentially lead to ambiguity by downstream decoders. Further studies are needed to understand how such ambiguity is resolved. We now focus on burst firing within the midbrain TS.

### Burst Firing in the Midbrain TS

**Figure 5:**
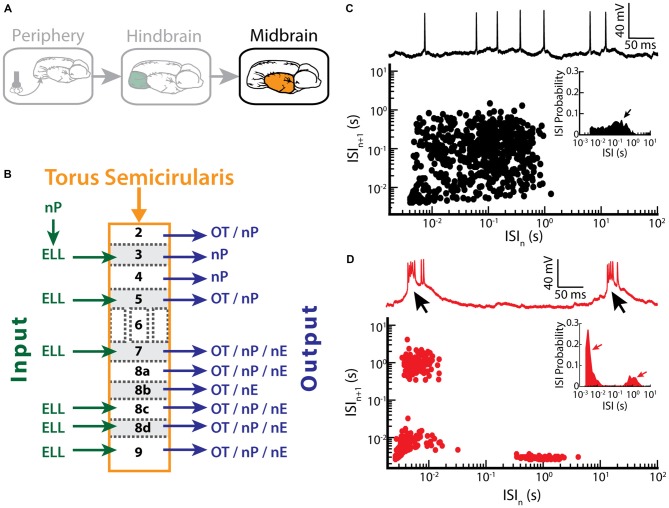
**Bursting in neurons in the midbrain TS. (A)** Neurons within the TS receive input from ELL pyramidal cells. **(B)** Summary of inputs to and outputs from TS layers. Note that TS layer 6 receives only input from the frequency modulation (FM) pathway that is not considered here. **(C)** Example recording of a non-bursting TS neuron (upper trace) and its return map (lower plot). The ISI distribution of this neuron shows a single peak (arrow) at around 100 ms (inset). **(D)** Example recording of a TS cell in bursting mode (upper trace). Arrows indicate the bursts of action potentials riding on top of a calcium spike. The return map displays clusters of dots close to the origin, the abscissa and the ordinate, indicating burst firing. *Inset*: the ISI distribution of this neuron shows two prominent peaks, as indicated by the arrows. Data plotted in **(C,D)** are from Chacron et al. (2009), Chacron and Fortune (2010), Khosravi-Hashemi et al. (2011), Khosravi-Hashemi and Chacron (2012).

ELL pyramidal cells project to the TS (Figure 5A), a midbrain structure which is homologous to the inferior colliculus in mammals (Chacron et al., 2011). The TS consists of 11 layers and contains about 50 cell types (Carr et al., 1981; Carr and Maler, 1985; Sproule et al., 2015). Most of these layers receive excitatory input from the ELL and in turn project to higher brain areas such as the optic tectum (OT), the nucleus praeeminentialis (nP) or the nucleus electrosensorius (nE; Figure 5B; Carr et al., 1981; Sproule et al., 2015).

Recent studies have shown that some TS neurons tend to fire bursts of action potentials while others tend to fire tonically (Khosravi-Hashemi et al., 2011; Khosravi-Hashemi and Chacron, 2012). Example tonic and bursting TS neurons with their ISI return maps and ISIHs are shown in Figures 5C,D, respectively. In particular, the ISI return map of the bursting TS neuron displays the characteristic L-shape (Figure 5D) and the ISIH is bimodal (Figure 5D, inset), which is similar to what was seen for ELL pyramidal cells after BAPTA treatment (compare with Figures 3F,G). The mechanism underlying burst firing in TS neurons involves T-type calcium channels (Chacron and Fortune, 2010). In general, T-type calcium channels are inactive at the neuron’s resting potential (~ −60 mV) and are de-inactivated by hyperpolarization to ~ −70 mV for about 100 ms. Once de-inactivated, a subsequent depolarization will lead to a subthreshold calcium spike, which further depolarizes the membrane and thus causes action potential firing (Figure 5D, black arrows). Manipulating the level of polarization of the membrane further revealed that bursting TS neurons display both burst and tonic modes of firing. The burst mode of firing is seen for more hyperpolarized levels whereas the tonic mode is obtained when sufficient depolarization is achieved by current injection through the electrode. As such, the mechanism underlying burst firing in TS neurons appears to be largely similar to that seen in thalamic relay neurons (Sherman, 1996, 2001). The functional role of bursting in TS neurons is discussed below.

**Figure 6:**
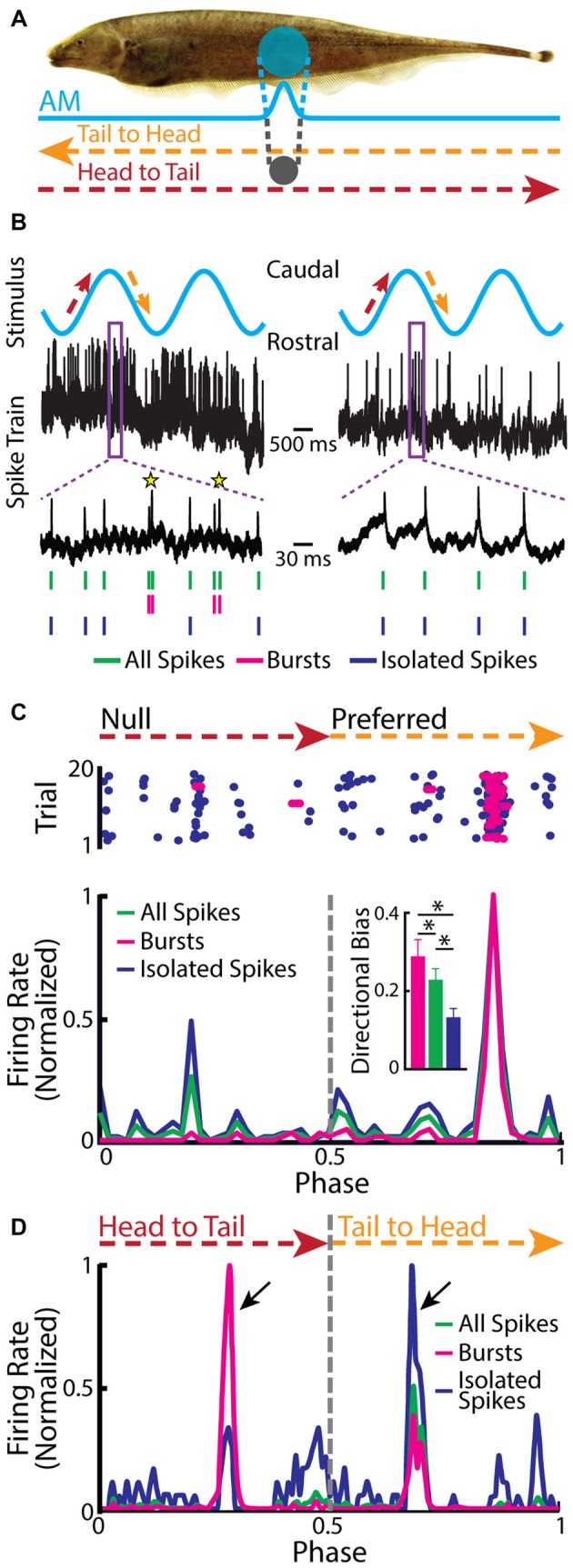
**Neurons in the midbrain TS respond to a moving object. (A)** Schematic showing the stimulation protocol. The gray sphere represents the moving object that was moved sinusoidally back and forth along the fish at a distance of about 1 cm lateral to the fish. The orange arrow indicates the tail-to-head direction, whereas the red arrow indicates the head-to-tail direction. The resulting local EOD AM and the spread of the electric image projected onto the skin are shown in blue. **(B)** Example *in vivo* recordings from a bursting TS (left) and a non-bursting TS neuron (right) to a moving object. Action potentials (green ticks) with ISIs that were shorter than the burst threshold were identified as belonging to bursts (magenta ticks), whereas those that were not were identified as isolated spikes (blue ticks). Burst events are indicated as yellow stars. **(C)** Raster plot from an example directionally selective bursting TS neuron. The spikes that belong to bursts are shown in magenta, whereas isolated spikes are shown in blue. *Bottom*: normalized PSTH for this same neuron computed from all spikes (both bursts and isolated spikes, green line), bursts (magenta line), and isolated spikes (blue line). *Inset*: population-averaged directional biases obtained for bursts (magenta), all spikes (green), and isolated spikes (blue). Asterisks indicate statistical significance at the *p* = 0.05 level using a signed-rank test. **(D)** Normalized PSTH for an example neuron where bursts and isolated spikes code for opposite directions of movement (arrows) computed from all spikes (green line), bursts (magenta line), and isolated spikes (blue line). The curves have been normalized by their maximum values. Directional bias values were 0.6, 0.5, and −0.63 for burst, all spikes, and isolated spikes, respectively. Data plotted in **(B–D)** are from Chacron et al. (2009), Chacron and Fortune (2010), Khosravi-Hashemi et al. (2011), Khosravi-Hashemi and Chacron (2012).

Recent studies have shown that burst firing within TS has different functional roles than those found for ELL pyramidal cells. This is because some TS neurons display directionally selective responses to an object that is moving laterally along the body of the fish (Figure 6A; Chacron and Fortune, 2010; Khosravi-Hashemi et al., 2011; Khosravi-Hashemi and Chacron, 2012). In contrast, ELL pyramidal cells do not display directional selectivity as mentioned above. Directional selectivity is observed when a neuron responds more strongly to an object moving in a given direction (i.e., the “preferred” direction) but responds weakly or not at all to the same object moving in the opposite direction (i.e., the “null” direction), and is a critical computation achieved in most brain circuits (Hubel and Wiesel, 1962; Borst and Egelhaaf, 1989, 1990; Jagadeesh et al., 1997; Srinivasan et al., 1999; Euler et al., 2002; Haag et al., 2004; Priebe and Ferster, 2008). The canonical model of directional selectivity is the so-called Reichardt detector, which requires at least two fundamental operations: the first is asymmetric filtering of information from at least two spatial locations within the receptive field, while the second is the nonlinear integration of these inputs (Reichardt, 1969, 1987; Borst and Helmstaedter, 2015). Studies of directional selectivity have typically compared the total number of spikes elicited by each direction of movement separately and without taking the specific temporal patterns of action potentials, such as bursts or isolated spikes, into account.

Closer examination of directionally selective TS neural responses to moving objects has revealed that there is strong burst firing when the object moves in the preferred direction (Figure 6B, left panel, Figure 6C) but much less or none when the object moves in the null direction (Figure 6B, right panel, Figure 6C). As such, the directional bias (i.e., the normalized difference between the response to the preferred direction and the response to the null direction) was significantly higher for the burst spikes than for the full spike train or the isolated spike train (Figure 6C, inset). In a small fraction of TS neurons, bursts and isolated spikes showed opposite directional biases (Khosravi-Hashemi and Chacron, 2012). As such, the directional bias computed from either the burst or isolated spike trains was larger in magnitude than that computed from the full spike train (Figure 6D).

It is likely that burst firing in TS neurons serves other functions as well, such as signaling the presence of a natural communication signal (Vonderschen and Chacron, 2011; Metzen et al., 2016), or encoding envelopes (McGillivray et al., 2012), but this has not been systematically investigated to date. Thus, to conclude this section, the functional roles of burst firing in TS have only just begun to be investigated. Of interest is the finding that burst firing can enhance the fidelity of signaling motion direction in TS neurons, which is similar to a previously proposed function that bursts will more reliably signal the presence of specific stimulus features because they are harder to elicit than single action potentials (Lisman, 1997). Moreover, the fact that bursts and isolated spikes can each encode opposite directions of movement in TS neurons is reminiscent of the parallel coding of the low and high frequency components of the stimulus by bursts and isolated spikes in ELL pyramidal cells. This suggests that parallel coding of different stimulus features by bursts and isolated spikes is a conserved function across multiple stages of processing in the electrosensory system. We next provide further evidence supporting this hypothesis by re-analyzing previously published data on EAs.

### A New Functional Role for Envelope Coding by Bursts and Isolated Spikes in EAs

Recent studies have shown that EAs can encode envelopes both at the single neuron (Savard et al., 2011; Metzen and Chacron, 2015) and the population levels (Metzen et al., 2015a,b). In particular, single EAs encode the time-varying envelope through changes in firing rate because of static nonlinearities such as rectification (i.e., the firing rate cannot be negative) and saturation (i.e., the firing rate cannot exceed the inverse of the absolute refractory period). Interestingly, responses to envelopes were either in phase or out of phase, revealing two distinct subpopulations of EAs (Metzen and Chacron, 2015). Further investigation revealed that the EA firing probability (i.e., the ratio of the firing rate to its maximum value which is given by the inverse absolute refractory period) determined the response phase. Indeed, the response was in phase with the envelope for EAs with firing probability less than 0.5 (i.e., “low” firing probability). In contrast, the response was out of phase with the envelope for EAs with firing probability greater than 0.5 (i.e., “high” firing probability). EAs whose firing probability was around 0.5 (i.e., “intermediate” firing probability) displayed responses that were either in phase or out of phase with the envelope but were significantly weaker than those of EAs with low or high firing probability (Metzen and Chacron, 2015). However, this previous study only considered the entire spike trains.

**Figure 7:**
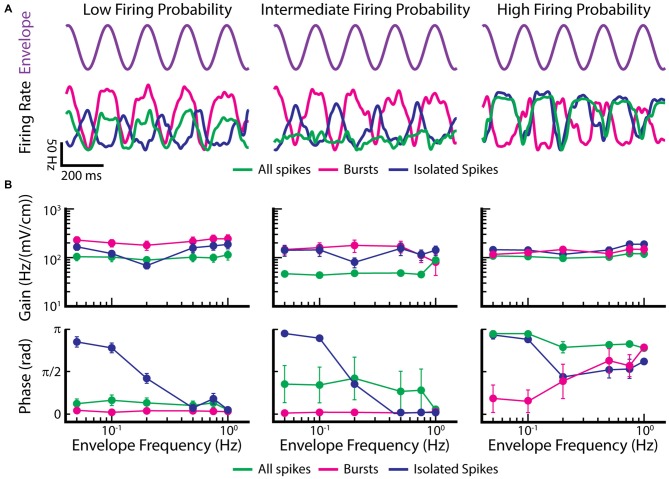
**Burst firing can improve the gain of EAs in response to envelopes. (A)** Example time dependent firing rates obtained for all spikes (green), bursts (magenta), and isolated spikes (blue) of EAs with a low firing probability (left), intermediate firing probability (middle) and high firing probability (right) to a sinusoidal envelope (top, purple). **(B)** Population-averaged gain (top) and phase (bottom) curves as a function of envelope frequency for EAs with low (left), intermediate (middle) and high (right) firing probabilities. Gain and phase curves for all spikes (green), bursts (magenta) and isolated spikes (blue) are shown. Data plotted in **(A,B)** are from Metzen and Chacron (2015).

We now investigate the coding properties of bursts and isolated spikes in response to envelopes. To do so, we used the previously published data from Metzen and Chacron (2015) and segregated bursts and isolated spikes using a burst threshold as described above for EAs. Figure 7 shows example time-dependent firing rates of EAs with low (Figure 7A, left), intermediate (Figure 7A, middle), and high firing probability (Figure 7A, right), in response to the envelope (Figure 7A, purple trace). Only considering the burst train in EAs displaying low and intermediate firing probabilities improves envelope coding as seen by higher gain values compared to when all spikes were taken into account (Figure 7B, magenta and green lines in left and middle top panel). When instead only considering isolated spikes, gain values were between those found for all spikes and bursts (Figure 7B, blue lines in left and middle top panel). In contrast, EAs displaying high firing probabilities gave qualitatively similar gain values for all three spike trains (i.e., the full spike train, the burst spike train, and the isolated spike train; Figure 7B, right panel). Interestingly, the filtered firing rate of the burst train was always in phase, whereas the firing rates for the isolated spike train was always out of phase with respect to the envelope for low frequencies (Figure 7B, bottom panels). These findings have important implications for decoding of envelope information by ELL pyramidal cells. Indeed, a recent study has shown that both ON- and OFF-type ELL pyramidal cell spike trains were largely in phase with the envelope (Huang et al., 2016), suggesting that they primarily decode information carried by bursts of action potentials in EAs. Further studies are however needed to test this hypothesis.

Thus, these findings demonstrate new functional roles for burst firing in EAs. The first is that bursts are more reliable indicators of changes in the envelope than the entire spike train in EAs with low and intermediate firing probabilities; this function is conceptually similar to that seen in TS neurons in response to moving objects. The second is that, for EAs with intermediate firing probability, bursts and isolated spikes respond in and out of phase with the envelope, respectively. This function is conceptually similar to that seen in ELL pyramidal cells and TS neurons, where bursts and isolated spikes are detecting different stimulus features. Such parallel coding by bursts and isolated spikes thus appears to be a general strategy that is found at multiple processing stages in the brain.

## Conclusion

In this review, we provided an overview about burst firing in the electrosensory system of weakly electric fish. We first gave insights in the mechanisms and then discussed functional aspects of burst firing occurring at successive processing stages from the periphery to the midbrain. Overall, the functional role of burst firing in the electrosensory system is to signal the presence of particular stimulus features such as the low-frequency components or natural electro-communication stimuli such as chirps. Bursts are normally harder to elicit than single action potentials and will thus more reliably signal the presence of their preferred feature: this was seen for both EAs in response to envelopes as well as TS neurons in response to moving objects. However, we have also seen that parallel coding by bursts and isolated spikes is manifested in EAs, ELL pyramidal cells, and TS neurons. While the stimuli considered were clearly different in all cases, the overall functional role remains conceptually similar. Such parallel coding by bursts and isolated spikes has also been observed in other systems. For example, in the mammalian thalamus, bursts and isolated spikes are also tuned to the low and high-frequency components of visual stimuli, respectively (Lesica and Stanley, 2004), similar to what was initially observed in ELL pyramidal cells (Oswald et al., 2004). Thus, the finding that burst firing has multiple functions across brain areas as well as for a given cell type is likely to be a general feature of neural processing.

## Author Contributions

MGM and MJC designed research. MGM performed research, MGM, RK, and MJC wrote the manuscript.

## Funding

This research was supported by the Fonds de Recherche du Québec—Nature et Technologies (RK, MJC), and the Canada Research Chairs (MJC).

## Conflict of Interest Statement

The authors declare that the research was conducted in the absence of any commercial or financial relationships that could be construed as a potential conflict of interest.
